# Sleepless but vigilant: unraveling the interplay of sleep loss and threat in response inhibition

**DOI:** 10.1093/sleep/zsaf008

**Published:** 2025-01-14

**Authors:** Johanna M Boardman, Jeryl Y L Lim

**Affiliations:** School of Nursing, Midwifery and Paramedicine, Australian Catholic University, Fitzroy, VIC, Australia; Turner Institute for Brain and Mental Health, Monash University, Melbourne, VIC, Australia; Turner Institute for Brain and Mental Health, Monash University, Melbourne, VIC, Australia

Cognitive control, which broadly encompasses a range of cognitive processes including inhibitory control, working memory, and cognitive flexibility, allows an individual to coordinate thoughts, update information, and modify behavior to achieve a specific goal [1, 2]. Cognitive control is suggested to be impaired by sleep loss [3, 4], but specific research on inhibitory control (the withholding of a response) particularly in an emotional context is lacking. A recent study by Nieuwenhuys et al. [5] investigated sleep loss effects on top-down inhibitory control under conditions of threat (a potential electric shock). The study utilized an Anticipatory Response Inhibition (ARI) task, administered under four sleep conditions, including one night of total sleep deprivation, two sleep restriction conditions (2 h and 4 h), and one well-rested, control condition (8 h). Extreme sleep loss (0 h and 2 h of sleep) slowed stopping speed, while the presence of threat led to faster stopping speeds. However, sleep loss did not exacerbate the effects of threat on performance, as was hypothesized. As such, the authors concluded sleep loss and threat influence response inhibition via distinct mechanisms. We agree with this conclusion and offer a discussion of supporting evidence.

In Nieuwenhuys et al. [5], threat was hypothesized to impair response inhibition (e.g. slow stopping speed) as increased attention to threat prioritizes threat related information, at the cost of goal directed control (e.g. top-down inhibitory control [6, 7]). Additionally, sleep loss was hypothesized to exacerbate the effect of threat of response inhibition. This hypothesis was largely driven by the notion sleep loss increases the hyper-limbic response to aversive and pleasure-evoking stimuli [8, 9]. Therefore, the combination of sleep loss and threat conditions was posited to impair response inhibition more than sleep or threat alone. However, results revealed threat instead improved response inhibition, a finding the authors attributed to increased motivation under threat conditions toward investing available cognitive resources in maintaining and improving task performance. Hence, one might expect threat to *mitigate* sleep-related impairments in response inhibition, contrary to the authors’ initial hypothesis. Intriguingly, this notion was not supported either, as indicated by nonsignificant sleep-by-threat interactions. Logically, and as briefly noted by the authors, this implies sleep loss and threat influence response inhibition through distinct mechanistic pathways. Here, we expand on this notion by proposing two supporting accounts.

First, the shock-induced threat condition in Nieuwenhuys et al. [5] likely activates the autonomic nervous system (ANS), which operates largely independently of top-down inhibitory control. Subcortical structures, such as the amygdala and brainstem, drive autonomic responses such as heightened vigilance and motor readiness, bypassing prefrontal regions susceptible to sleep loss and enhancing response inhibition by increasing attention to threat [10–12]. Thus, with little interplay between the ANS and top-down inhibitory control, no compounding or mitigating effect would produce a significant interaction. Notably, this also highlights a distinction between physiologic threats (e.g. shock, burn) and threats of a more “cognitive” nature, such as the prospect of financial loss. Unlike physiologic threats, which directly activate the ANS and its reflexive pathways, cognitive threats may engage top-down inhibitory control to a greater extent. Indeed, it would be interesting to examine whether a sleep-by-threat interaction might emerge under conditions involving cognitive threats, hence providing more nuanced insights into how different types of threats influence response inhibition under sleep-deprived states.

Second, we note the ARI task used in Nieuwenhuys et al. demands greater fine motor control compared with conventional Go/No-Go/Stop Signal tasks, as it combines reaction time with accuracy in hitting a target line on Go trials. This raises concerns around whether observed effects on response inhibition stem from changes in context monitoring (the detection and interpretation of task-relevant signals), motoric stopping (halting the ongoing response), or a combination of both. Previously, Chatham et al. [13] provides evidence for a dissociation between these two processes in response inhibition. Specifically, activity in the right ventrolateral prefrontal cortex, traditionally thought to reflect motoric stopping, was similar across two tasks differing in motoric stopping requirements. Furthermore, EEG evidence in Chatham et al. [13] indicated the “Stop P3” signal, also previously thought to reflect motoric stopping, was observed to be larger when the task only required context monitoring and not motoric stopping. This implies that context monitoring demands are more cognitively taxing than motoric stopping and highlights the importance of disentangling these components when interpreting effects on response inhibition. In Nieuwenhuys et al. [5], shorter stop-signal delays (SSDs) observed in the threat condition suggest performance improvements, but it remains unclear whether these reflect enhanced context monitoring, such as heightened attention to signals, or improvements in halting motor responses. Similarly, the absence of significant sleep-by-threat interactions on SSDs alone does not offer clarity into how sleep loss and threat might differentially affect both cognitive and motor components, or whether an interplay between these effects could have resulted in no observable interaction. Hence, to better identify components of response inhibition impacted by sleep loss or threat, it is crucial for future studies to replicate the design of Nieuwenhuys et al. [5] with accompanying neuroimaging, EEG, or pupillometry data.

Nieuwenhuys et al. [5] are the first to examine extreme sleep loss effects on response inhibition under threat conditions. Considerable strengths of the study include the use of multiple “doses” of sleep loss, and the administration of an ARI task, which is considered a more accurate measure of response inhibition as a form of top-down inhibitory control over a traditional Go/Nogo task. That being said, it is important to also consider the generalizability of the findings to real-world contexts. Nieuwenhuys et al. [5] showed response inhibition deficits only at very severe levels of sleep deprivation, using a task that minimized complexity to constrain the scope of cognitive domains being implicated, as is typical of most cognitive tasks. Sleep loss may paradoxically impair simple tasks more than complex ones [14]. This is attributable to the engaging nature of complex tasks, which demand greater effort and allow individuals to compensate for sleep loss. Conversely, simple and monotonous tasks may induce boredom and exacerbate performance deficits [14]. Therefore, given the relatively simple nature of the ARI task utilized by Nieuwenhuys et al. [4], sleep loss effects may not extend to tasks which are more cognitively demanding, and arguably, more reflective of real-world scenarios. To better understand how sleep loss and threat may affect real-world behavior, tasks that accurately capture inhibitory control within a complex context (e.g. driving) are needed in future research. Nonetheless, Nieuwenhuys et al. [4] have provided an intriguing initial investigation into sleep and threat effects on response inhibition. Future studies replicating their approach with the addition of neuroimaging, EEG, and/or pupillometry are a critical next step in unraveling the complex interplay of sleep and threat on response inhibition.
